# The Simultaneous Formation of Acrylamide, β-carbolines, and Advanced Glycation End Products in a Chemical Model System: Effect of Multiple Precursor Amino Acids

**DOI:** 10.3389/fnut.2022.852717

**Published:** 2022-03-09

**Authors:** Cuyu Chen, Ye Jiao, Maomao Zeng, Zhiyong He, Qingwu Shen, Jie Chen, Wei Quan

**Affiliations:** ^1^College of Food Science and Technology, Hunan Agricultural University, Changsha, China; ^2^School of Chemistry and Food Engineering, Changsha University of Science and Technology, Changsha, China; ^3^State Key Laboratory of Food Science and Technology, Jiangnan University, Wuxi, China; ^4^International Joint Laboratory on Food Safety, Jiangnan University, Wuxi, China

**Keywords:** precursor amino acids, acrylamide, N^ε^-(carboxymethyl)lysine, N^ε^-(carboxyethyl)lysine, β-carboline heterocyclic amines

## Abstract

This study investigated the effect of multiple precursor amino acids on the simultaneous formation of acrylamide, β-carbolines (i. e., harmane and norharmane), and advanced glycation end products (AGEs) [i.e., N^ε^-(carboxymethyl)lysine and N^ε^-(carboxyethyl)lysine] via a chemical model system. This model system was established with single or multiple precursor amino acids, including lysine–glucose (Lys/Glu), asparagine–glucose (Asn/Glu), tryptophan–glucose (Trp/Glu), and a combination of these amino acids (Com/Glu). Kinetic parameters were calculated by multiresponse non-linear regression models. We found that the *k* values of the AGEs and of acrylamide decreased, while those of harmane increased in the Com/Glu model when heated to 170 and 200°C. Our results indicated that the precursor amino acid of acrylamide and AGEs compete for α-dicarbonyl compounds, leading to a decrease in the formation of AGEs and acrylamide. Moreover, compared with asparagine, the precursor amino acid of β-carbolines was more likely to react with acetaldehyde by Pictet–Spengler condensation, which increased the formation of harmane and decreased the formation of acrylamide via the acrolein pathway.

## Introduction

The Maillard reaction (MR) refers to the interaction between protein's free amine groups and carbohydrate's carbonyl groups (1, 2) and is often used in food processing to imbue food products with appealing flavors and colors. Incidentally, MR also produces some undesirable and harmful products that can have a negative impact on human's health (1), such as heterocyclic amines, advanced glycation end products, and acrylamide. These products, produced in heat-processed foods, have aroused widespread concern because of their carcinogenic, mutagenic, and other health risks.

Acrylamide is major harmful products of MR, which was first identified in high-temperature–processed, starch-rich food including french fries, potato chips, coffee, and bakery products (3). Currently, enough evidence has led acrylamide to be classified as a human carcinogen and a neurotoxic compound that exerts toxic effects on the nervous system, liver, and kidneys (4). In general, according to the literature, the formation of acrylamide has been reported to proceed in a step-wise manner: A Schiff base is first formed in the MR's early stages, and then it rearranges to produce an Amadori/Heyns product that can be decarboxylated to form acrylamide directly or reacted with asparagine (Asn) to form acrylamide via a Strecker degradation (5–7). In addition, the acrolein pathway is another important way to produce acrylamide, and acrolein is usually generated by the oxidation of ethanal during the MR (7, 8).

Advanced glycation end products (AGEs) refer to a group of structurally complex and chemically stable Maillard reaction harmful products (MRHPs) generated in the last stage of the MR (9). N^ε^-(carboxymethyl)lysine (CML) and N^ε^-(carboxyethyl)lysine (CEL) are typical AGEs found in food including tea, milk, meat, and bread (10, 11). In brief, CML is formed by the reaction of a reactive α-dicarbonyl, including glyoxal (GO), and CEL is produced by the reaction of methylglyoxal (MGO) with lysine (Lys) in the MR (12, 13). Diets rich in AGEs may increase the potential risk for several chronic kidney and liver diseases, diabetes, cardiovascular disease, and Alzheimer's disease (9, 12).

β-carboline heterocyclic amines (HAs) including harmane and norharmane are a series of complex food carcinogens and mutagens, which can form at lower temperatures without the presence of creatine and creatinine (14, 15). Toxicology studies have indicated that harmane and norharmane display comutagenic activity in the presence of other carcinogens, and they also show neurotoxic characteristics, which may lead to the development of some nervous system disorders (16). As studies have shown (17), except for the dehydration and β-elimination of AP (15), the oxidation of tetrahydro-β-carbolines (THβCs) is also an important way to produce β-carboline HAs, although THβCs are usually generated through the Pictet–Spengler condensation between tryptophan (Trp) and acetaldehyde during the MR (14, 15, 17).

Until now, although a large number of studies have focused on formation pathways, the influencing factors and inhibition methods for the individual harmful compounds or homologs with similar structure. The formation mechanisms of the above-mentioned MRHPs have only been investigated using MR chemical model systems with a single substrate amino acid and a single reducing sugar (18, 19). These chemical model systems ignore the fact that some precursors for the formation of these MRHPs, including Lys, Asn, and Trp, exist together in certain foods such as potatoes, cookies, and bread (20). Studies that have focused on the effect of multiple precursor amino acids on the simultaneous generation of these MRHPs in heat-processed food are few. Although some studies have indicated that the addition of amino acids including lysine and tryptophan were effective at inhibiting the formation of acrylamide in MR chemical model systems, the formation of AGEs and β-carboline HAs was not considered (21, 22). Moreover, in cereals and potato-based food, the amount of reducing sugars is relatively lower than the amount of free amino acids, and the ratio of each amino acid is different (20). Because of the limitations of reducing sugars, lysine, asparagine, and tryptophan may have to compete to participate in the MR, and there are some similar intermediate compounds in the formation of MRHPs. The effect of multiple precursor amino acids on the formation of the previously mentioned MRHPs remains unclear.

Based on an aqueous MR chemical model system, the effect of multiple precursor amino acids including Lys, Asn, and Trp on the simultaneous formation of acrylamide, β-carboline HAs, and AGEs was investigated. The present study offers insight into the reaction mechanism and provides mathematical evidence for predicting the simultaneous formation of acrylamide, β-carboline HAs (harmane and norharmane), and AGEs (CML and CEL) in high-temperature-processed cereals and potato-based foods.

## Materials and Methods

### Chemicals

The standards of tryptophan, and asparagine, acetaldehyde, acrolein, glyoxal and methylglyoxal, and nonafluoropentanoic acid were purchased from J&K Scientific Co, Ltd. (Beijing, China). harmane, norharman, *N*^ε^-(carboxymethyl)lysine (CML), d_4_-CML, *N*^ε^-(carboxyethyl)lysine (CEL), and d_4_-CEL were obtained by Santa Cruz Biotechnology Co. (Paso Robles, CA). Glucose and ^13^C_6_-glucose, lysine and d_4_-lysine, acrylamide and ^13^C_3_-acrylamide were purchased from Sigma-Aldrich (Darmstadlt, Germany). Other amalytical grade chemicals were purchased from Sinopharm Chemical Reagent Co, Ltd. (Shanghai, China).

### Preparation of Maillard Reaction Model Systems and Thermal Reaction

According to previous studies with some modifications (23, 24), aqueous Maillard reaction model systems were prepared in phosphate buffer (0.1 M, pH 6.8). Four amino acid–glucose reaction solutions containing Lys–glucose (Lys/Glu; 30/100 mmol), Asn–glucose (Asn/Glu; 200/100 mmol), Trp–glucose (Trp/Glu; 5/100 mmol), or a combination of Lys–Asn–Trp–glucose (Com/Glu; 100/200/5/100 mmol).The ratios and concentrations of amino acids and glucose in the chemical model system (Lys 30, Asn 200, Trp 5, glucose 100 mmol) were selected based on their actual proportions in real cereal and potato-based food (20, 25, 26), and their concentrations were appropriately magnified to better evaluate the intermediates and final products.

Next, 20 ml reaction solutions were added to glass reaction vials with Teflon caps. The reaction vials were fully sealed as much as possible to avoid water evaporation and oil absorption. The reaction vials were heated at 170 and 200°C for 0–21 min using a Jintan thermostat-controlled oil bath (Zhejiang, China). Each glass reaction vial was preheated for 5 min before sealing and inserting it into the oil bath. The reaction vials were taken out of the oil bath at predetermined heating times (3, 6, 9, 12, 15, 18, and 21 min), cooled in an ice bath to stop any further reaction, and stored at −80°C for further analysis.

### Determination of Reactants

After centrifugation and dilution, the 300 μl reaction sample were added to 300 μl of an internal standard containing 100 μg/ml d_4_-lysine, and 200 μg/ml ^13^C_6_-glucose.

The identification and quantification of lysine, asparagine, tryptophan, and glucose were conducted on an Acquity UHPLC system equipped with a triple quadrupole MS (Waters, Milford, MA, USA). The separation of analytes was performed on a Waters Atlantics dC18 column (250 × 4.6 mm i.d, 3.0 μm) using a gradient elution with 0.1% formic acid (pH 6.8) (solvent A) and acetonitrile (solvent B). The solvent composition was 0–0.1 min, 5% A; 0.1–10 min, 5–100% A; 10–10.5 min, 100–5% A; and 10.5%−15 min, 5% A. The flow rate was 0.3 mL/min, the column temperature was 30°C and the injection volume was 10 μL. The capillary voltage was 3.5 kV. The temperature of the ion source and that of the desolvation gas were 130 and 350°C, respectively. The fragment used for quantification were *m/z* 133 → 74 (asparagine); *m/z* 147 → 84 (lysine); *m/z* 151 → 88 (d_4_-lysine); *m/z* 151 → 88 (d_4_-lysine); *m/z* 383 → 203 (glucose); and *m/z* 395 → 209 (^13^C_6_-glucose).

### Determination of Intermediate MRPs

#### Glyoxal and Methylglyoxal

Analysis of α-dicarbonyl compounds including glyoxal (GO) and methylglyoxal (MGO) was carried out as described previously (27). Reaction samples were centrifuged at 12,000 g for 5 min, then 300 μl supernatant was collected used for derivatization. Derivatization of α-dicarbonyl compounds was carried out with 150 μl of 5 μM *o*-phenylenediamine. In addition, 100 μl of 2,3-hexanedione (4 μM) was added as an internal standard. Then the mixture was kept at 4°C in the dark for 12 h.

The derivatives of α-dicarbonyl compounds were determined by using a Waters 2695 LC system with a Micromass Quattro Micro triple quadrupole MS and Waters X-Bridge C18 column (2.1 × 100 mm, 3.5 μm, Milford, MA, USA) at a flow rate of 0.3 mL/min. The gradient mixture was started from 30% A and increased to 90% and 100% A in 5 and 3 min, respectively. Then it decreased to 30% A in 2 min and the 30% A remained for 5 min, mobile phase A was 1% formic acid and B was methanol. The α-dicarbonyl compounds were identified with the same MS parameters used in the analysis for CML and CEL. The fragments used of the α-dicarbonyl derivatives were *m/z* 131 → 77 (GO), 145 → 77 (MGO), and 187 → 77 (internal standard).

#### Acetaldehyde and Acrolein

According to the method reported by Uchiyama et al. (28) with some modification, 3 ml samples or standard solutions of acetaldehyde or acrolein were mixed with 1.5 ml 2% NaCl−0.8 mol/L HCl solution, 1.0 ml 15% Na_2_HPO_4_, and 1.0 ml 0.2% 2,4-dinitrophenylhydrazine, added sequentially. Then the mixed solution was heated in a water bath at 65°C for 20 min. After cooling to room temperature, 5 ml hexane was added twice for extraction of acrolein. Then 2 ml hexane was filtered through a 0.22-μm organic microporous membrane for HPLC analysis. Lichrospher C18 (4.6 × 250 mm, 5.0 μm) column was selected for HPLC separation. For each sample, 10 μL was injected onto the column and allowed to elute isocratically at 30°C with 62% acetonitrile in water at a flow rate of 1.0 mL/min. The detection walvelength was 365 nm. A run time of 20 min was enough for complete elution of sample components.

### Determination of Final MRPs

#### Melanoidins

The concentrations of melanoidins were determined at 470 nm on a spectrophotometer (UV-1601, Shimadzu, Kyoto, Japan). The concentrations of melanoidins were calculated from the Lambert–Beer equation with an extinction coefficient of 282 L/mol·cm, a value derived for melanoidins formed from glucose and asparagine (24, 29).

#### CML and CEL

According to Jiao et al. (10) and Yu et al. (11), 150-μl reaction sample was spiked with 150 μL of internal standard solution which containing 71.2 ng of d_4_-CML and 66.0 ng of d_4_-CEL. Chromatographic separation was performed on a Waters 2695 liuid chromatograph (LC) module coupled with a Waters X-Bridge C18 column (2.1 × 100 mm, 3.5 μm, Milford, MA, USA). The mobile phase A was acetonitrile and mobile phase B was NFPA (5 mM). The flow rate was 0.3 mL/min, the injection volume was 5 μL and the column temperature was set to 40°C. The gradient elution was performed as follows: 0–0.1 min, 5% A; 0.1–5 min, 5–60% A; 5–8 min, 60%−100% A; 8–10 min, 100% A; 10–12 min, 100–5% A; 12–20 min, 5% A.

The LC was interfaced with a Micromass Quattro Micro triple quadrupole mass spectrometer (Manchester, UK). The mass spectrometer (MS) was operated in an electrospray ionization (ESI) positive mode with multiple reaction monitoring (MRM). The capillary voltage was 3.6 kV. Temperatures of the ionization source and the desolvation gas were 110 and 400°C, respectively. The flow rates of the cone gas and desolvation were 50 and 600 L/h, respectively. Argon gas was used as the collision gas in the collision cell. For CML, d_4_-CM, CEL, and d_4_-CEL, the fragments *m/z* 205 → 84, *m/z* 209 → 88, *m/z* 219 → 84, and *m/z* 223 → 88, respectively, were used for quantification.

#### Acrylamide, Harmane, and Norharmane

After centrifugation and dilution, the 300 μl reaction sample were added to 300 μl of an internal standard containing 100 μg/ml ^13^C_3_-acrylamide. The identification and quantification of acrylamide, harman, and norharman were also conducted on an Acquity UHPLC system equipped with a triple quadrupole MS (Waters, Milford, MA, USA). The parameters of UHPLC-MS were same as section 2.3. The fragment used for quantification were *m/z* 72 → 55 (acrylamide); *m/z* 75 → 58 (^13^C_3_-acrylamide); *m/z* 169 → 115 (norharman).

### Kinetic Modeling

**Figure 1:**
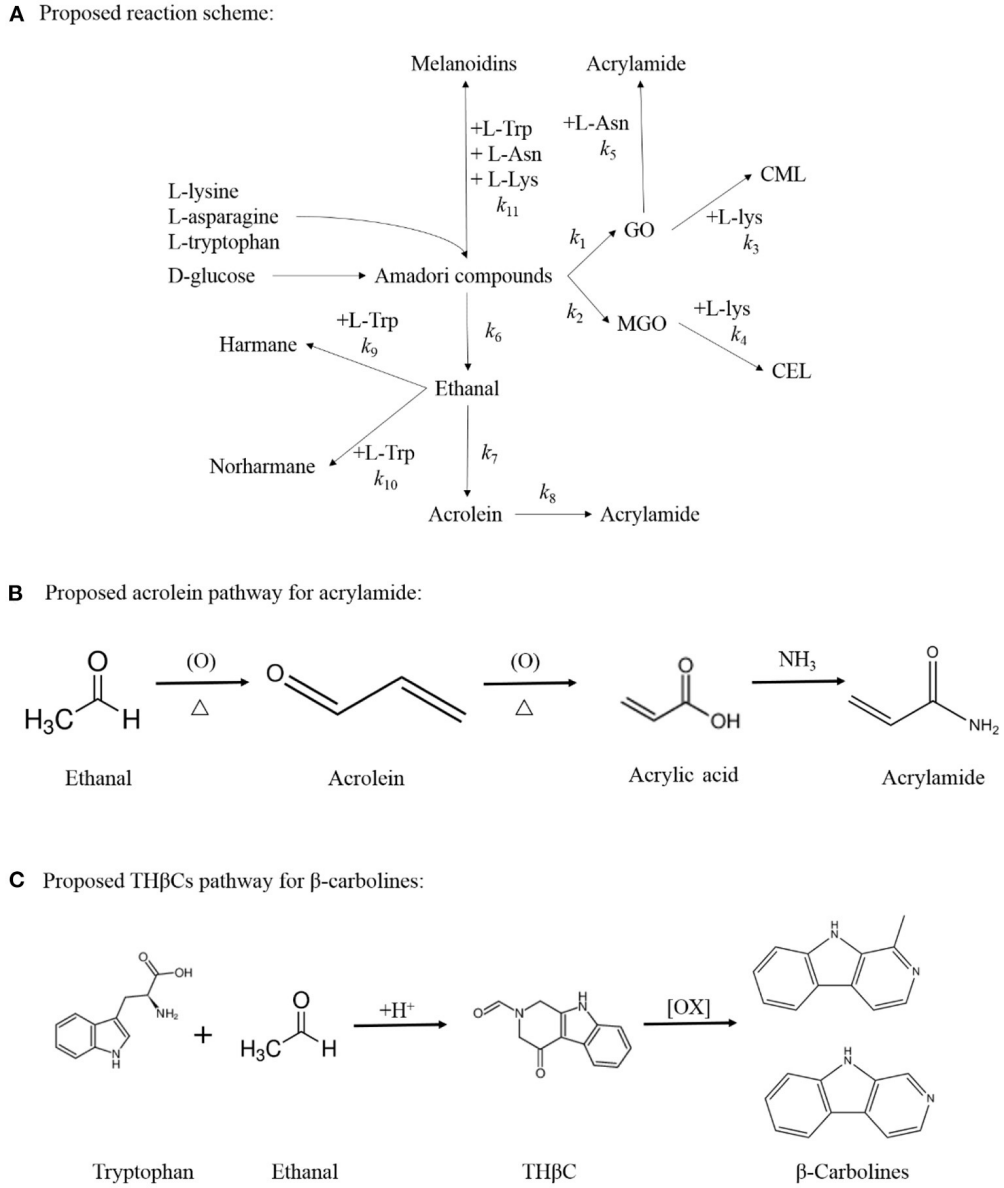
Proposed reaction scheme for the simultaneous formation of acrylamide, CML, CEL, harman and norharman in multiple precursors amino acids/ glucose chemical model system **(A)**. Proposed acrolein pathway for the production of acrylamide **(B)** and Proposed THβC pathway for the generation of β-carbolines **(C)**.

In the present study, only GO, MGO, and acrolein was detected and taken into consideration during the kinetic modeling. The kinetic model for the MR model system was developed based on the proposed reaction pathways as shown in Figure 1A. Equations for the reaction rates of each compound are expressed in Supplementary Figure 1. For modeling purposes, the average concentrations of analysis were used (measured in triplicate), and data were normalized and expressed in millimoles per liter. All experiment data were made to fit the proposed kinetic model, and the kinetic parameters (*k*) were estimated based on the non-linear curve fitting function of the Origin 8.5 software (OriginLab, USA) and SAS 9.0 software (SAS Institute Inc, China). Moreover, the R-squared (*R*^2^) values were calculated to determine the fitting performance of the non-linear fitted models (29, 30).

### Statistical Analysis

Analysis of all components was conducted in triplicate, with values reported as the mean ± standard deviation of three independent experiments. Statistical analysis was carried out using the general linear model procedure of Statistix software 9.0 (Analytical Software, Tallahassee, FL, USA). Significant differences (*p* < 0.05) between means were identified by the least significance difference procedure.

## Results and Discussion

### The Concentration Changes of Reaction Substrates in the MR Model System

**Figure 2:**
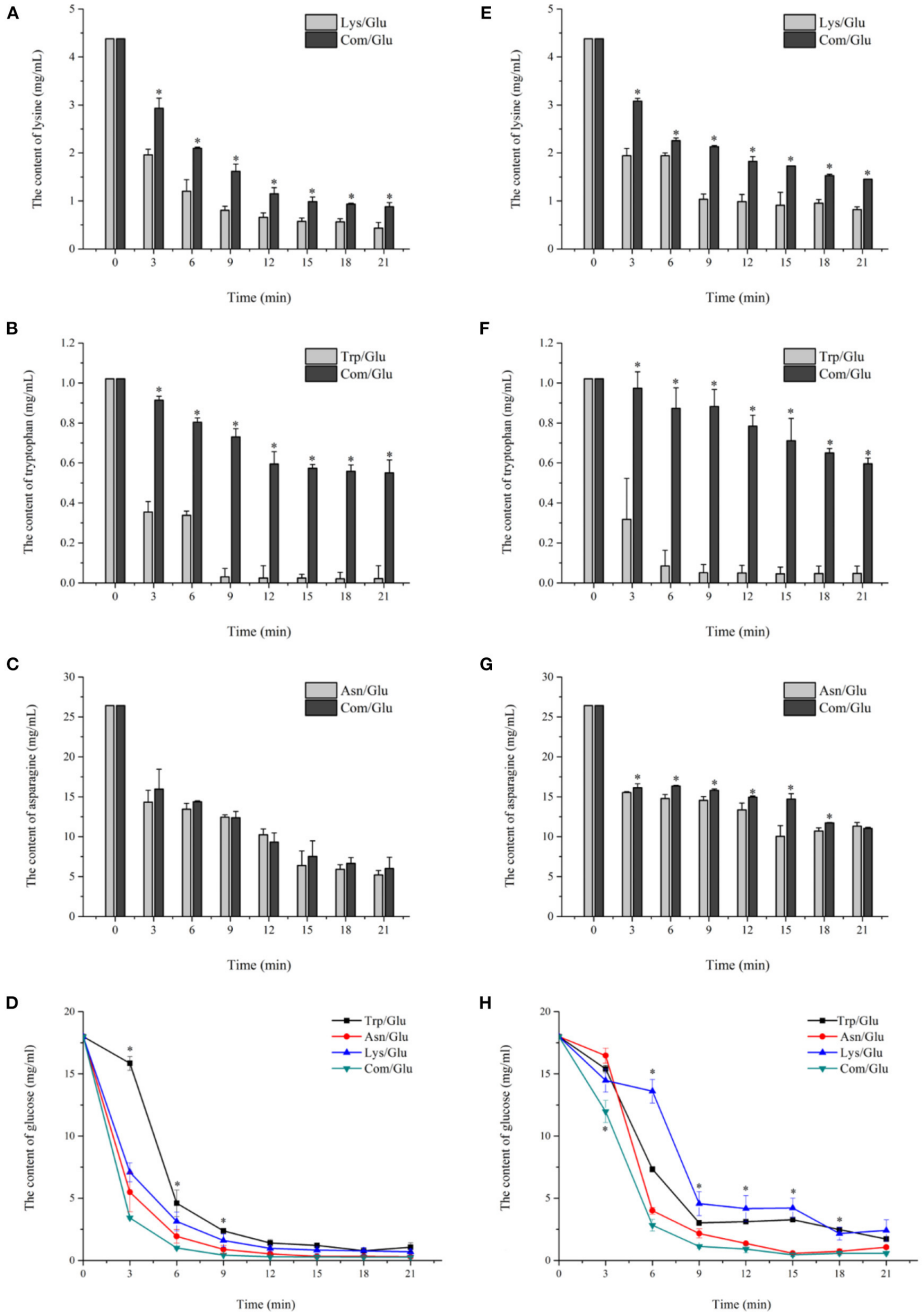
Kinetic profiles of Lys **(A,E)**, Trp **(B,F)**, Asn **(C,G)**, and glucose **(D,H)** in the different amino acid/glucose model system heated at 170 °C **(A–D)** and 200 °C **(E–H)**. Data were expressed as mean ± SD in triplicates (*n* = 3), *, (*P* < 0.05).

As shown in Figure 2, the concentrations of lysine, asparagine, tryptophan, and glucose declined with increased heating time; the degradation rates of amino acids and glucose also decreased over time, which is consistent with previously reported results (7). The degradation rates of the majority of the amino acids as well as glucose underwent a steep decline during the initial 9 min of heating. When heated to 170°C (Figures 2A–D), the concentration of Trp in Trp/Glu model decreased by 96.9% at 9 min compared with the initial Trp concentration. Lys showed results similar to those for Trp, with a reduction of 83.1% at 9 min in the Lys/Glu model, followed by a gradual reduction in the decrease rate. At the final stage of the reaction, the concentrations of both Trp and Lys in the control group were nearly 0 after 21 min of applied heat, as expected. However, Trp and Lys levels in the Com/Glu model remained at 58.4 and 21.5% of their initial concentrations, respectively. Unlike Trp and Lys, Asn was reduced by 80.3 and 77.3% in the Asn/Glu and Com/Glu models, respectively, indicating that the degradation rates were the same in both the Asn/Glu and Com/Glu models. Figures 2E–H shows the concentration change of the amino acids at 200°C, which was similar to their kinetics at 170°C. The reduction of the amino acids in the Com/Glu model was also significantly less than that of the single amino acid/Glu model.

The concentration of another important MR precursor, glucose, was also monitored (Figures 2D,H). When heated at 170°C for 9 min (Figure 2D), the glucose concentration in the Asn/Glu, Trp/Glu, Lys/Glu, and Com/Glu models decreased by 95.1, 86.8, 91.1, and 97.6%, respectively, and the gradually lessening reduction rate of glucose eventually decreased by 98.3, 94.0, 96.1, and 98.5%, respectively. Meanwhile, similar results were obtained at 200°C (Figure 2H). At 200°C, the concentration of glucose in the Asn/Glu, Trp/Glu, Lys/Glu, and Com/Glu models decreased by 88.9, 83.2, 74.6, and 94.6%, respectively, by 9 min, and had decreased by 94.7, 90.4, 97.7, and 83.8%, respectively, at the end of heating. Comparing the different model systems, the reduction of glucose in the Trp/Glu and Lys/Glu models was less than the reduction of glucose in the Asn/Glu and Com/Glu models.

### The Concentration Changes of Reaction Intermediates in the MR Model System

In order to further analyze the generation mechanism of related MRHPs in the model system, several intermediate compounds were selected by reference to the literature (5, 6, 13). Changes in the concentration of these compounds during the reactions were determined.

**Figure 3:**
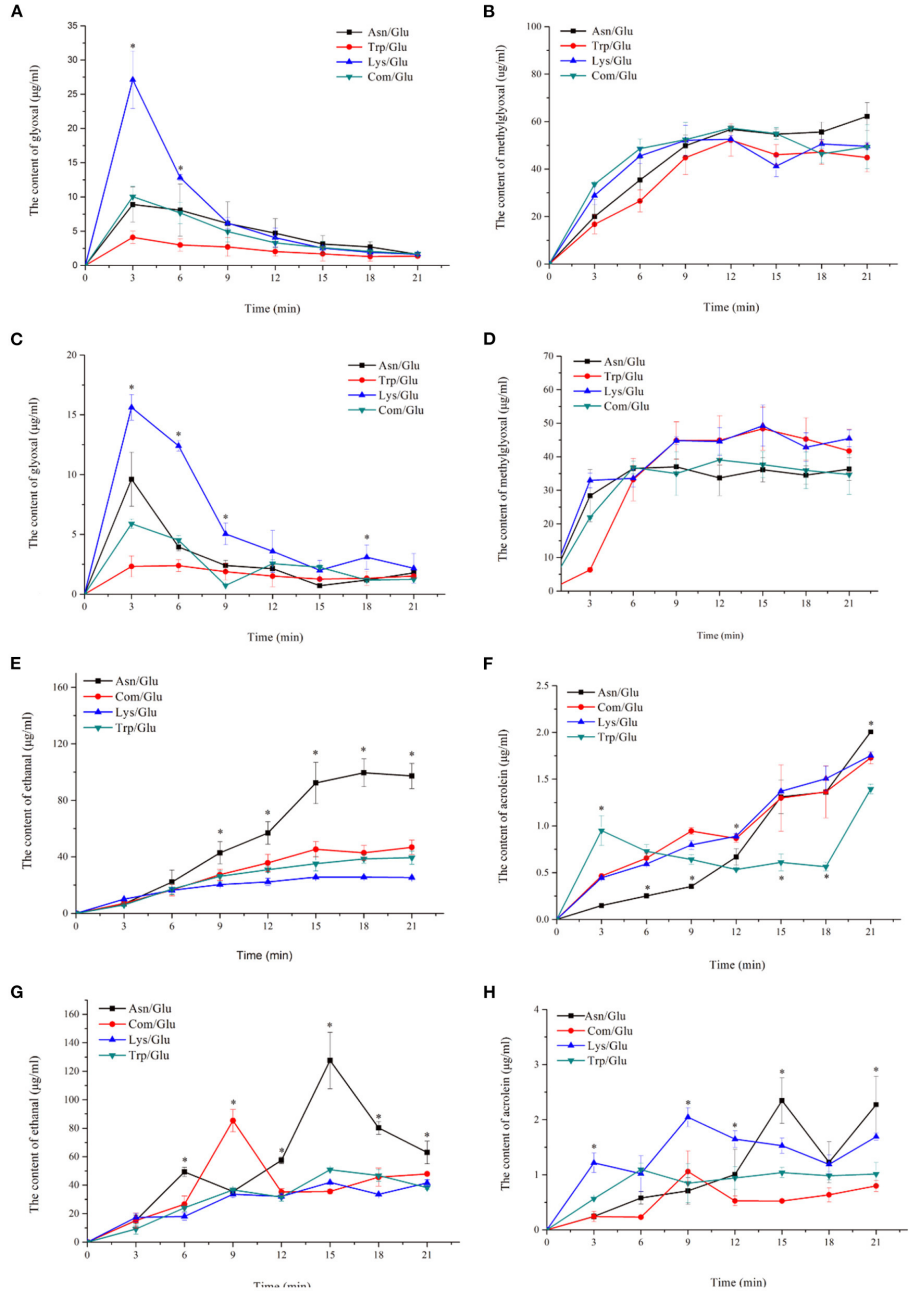
Kinetic profiles of major ketone and aldehyde intermediate compounds: glyoxal **(A,B)**, methylglyoxal **(C,D)**, acetaldehyde **(E,F)** and acrolein **(G,H)** in the different amino acid/glucose model system heated at 170°C **(A,C,E,G)** and 200°C **(B,D,F,H)**. Data were expressed as mean ± SD in triplicates (*n* = 3), *, (*P* < 0.05).

α-Dicarbonyl compounds are important intermediates formed in MRs. They are mainly produced by the thermal degradation of glucose and the decarboxylation of a Schiff base that reacts with amino acids to form a series of MR products (31). As Figures 3A,B shows, all of the model systems produced a large amount of GO at the initial stage of the reaction (after 3 min), but as the reaction progressed, the concentration of GO decreased. In addition, a higher GO content was observed at 170°C rather than at 200°C. Comparing the different model systems, the content of GO in the Trp/Glu model was significantly lower than in the other models, especially for the Lys/Glu model. Despite this, there was no significant difference in the GO content in the different model systems at the end of the reaction.

The changes in MGO concentration are shown in Figures 3C,D. The content of MGO reached a relatively high value at 9–15 min and then either remained at a stable level or decreased. Similar to the effects seen with the GO content, higher temperatures efficiently induced the formation of MGO. The generation of MGO in the different model systems was different than what was observed for GO, however. When heated to 170°C, the amount of MGO produced in the Asn/Glu model was higher than in the other systems. When heated to 200°C, the Lys/Glu model showed the highest content of MGO.

Aldehydes are also important MR intermediates and are degradation products of glucose at high temperatures (19). As shown in Figures 3E,F, the formation of ethanal was significantly different (*p* < 0.05) in the different model systems. The Asn/Glu model had a higher reactivity, which generated more ethanal than in the other models. The content of ethanal in the Asn/Glu model was 97.25 ± 8.91 μg/ml at 170°C and 62.97 ± 7.97 μg/ml at 200°C, which was 51.92–73.98% and 24.01–34.20% higher, respectively, than in the other model systems.

Acrolein is produced by the oxidation of ethanal, so it is an important intermediate compound that can make acrylamide through the acrolein pathway (7, 8, 32). The content of acrolein was significantly different (*p* < 0.05) in each of the model systems. At the two heating temperatures (Figures 3G,H), the concentration of acrolein formed in the Asn/Glu model system was the highest, while it was the lowest in the Trp/Glu and Com/Glu models.

### The Concentration Changes of MRHPs in the MR Model System

#### Acrylamide

**Figure 4:**
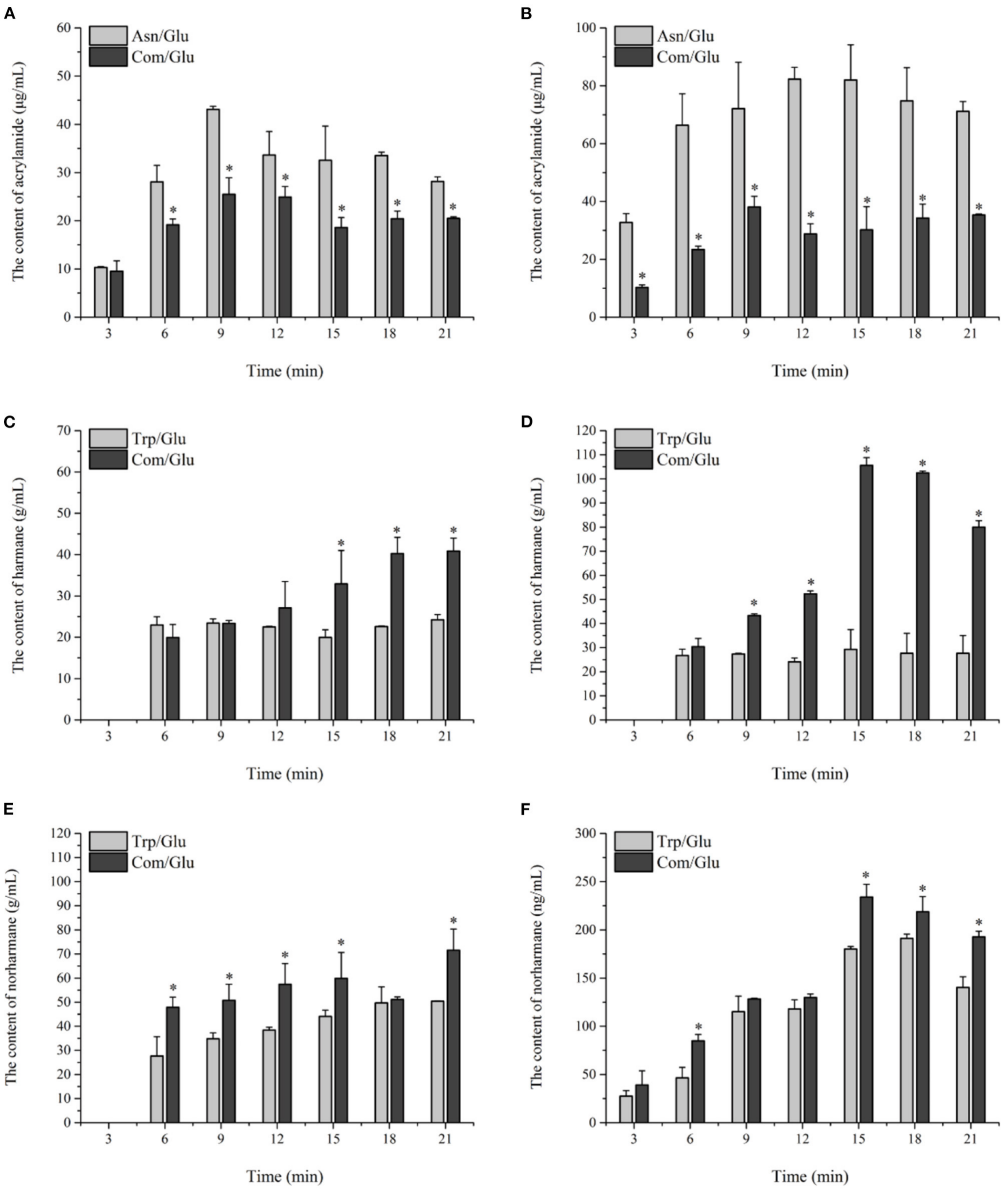
Kinetic profiles of Maillard reaction products includes Acrylamide **(A,B)**, harman **(C,D)**, and norharman **(E,F)** in the different amino acid/glucose model system heated at 170°C **(A,C,E)** and 200°C **(B,D,F)**. Data were expressed as mean ± SD in triplicates (*n* = 3), *, (*P* < 0.05).

The kinetics of acrylamide in the different models are shown in Figures 4A,B. Acrylamide concentrations in all the models underwent a stage during the initial 12 min of heating in which the generation of acrylamide was dominant. After prolonged heating, a decrease in the acrylamide content was observed, which can be attributed to acrylamide's elimination becoming dominant over acrylamide's formation (33). Since acrylamide is mainly reactive through its double bond and can react as an electrophile by a 1,4-addition to nucleophiles, the elimination of acrylamide is ascribed to acrylamide degradation, polymerization, or reactions with various other components present or formed in the model system (33). The content of acrylamide formed at 200°C was significantly higher than that at 170°C, which is similar to the results found by Bråthen et al. (32) and Rydberg et al. (34) who showed that the a maximum amount of acrylamide was formed at approximately 200°C. The acrylamide content in the Asn/Glu model reached a maximum of 43.12 ± 0.62 μg/ml and 82.28 ± 4.01 μg/ml and then was reduced by 34.7 and 13.5% at 170 and 200°C, respectively. Trp and Lys may reduce the concentration of acrylamide as Koutsidis et al. (22) reported. The maximum content of acrylamide in the Com/Glu model reached only 25.51 ± 3.42 μg/ml at 170°C and 38.05 ± 3.71 μg/ml at 200°C, which was significantly lower (*p* < 0.05) than the results seen in the Asn/Glu model.

#### Harmane and Norharmane

In general, the contents of harmane and norharmane increased with time when heated to 170°C (Figures 4C,E). The highest concentration of harmane and norharmane in the Trp/Glu and Com/Glu models was obvious at 21 min. When heated to 200°C (Figures 4D,F), the concentrations of harmane and norharmane were significantly reduced after reaching a maximum value. Furthermore, samples heated to 200°C showed a higher content of harmane and norharmane than the samples heated to 170°C, which is consistent with previous results (15). However, it should be noted that when comparing the content of harmane in the Trp/Glu and Com/Glu models, the amount of harmane produced in the Com/Glu model was significantly higher (*p* < 0.05) than that in the Trp/Glu model.

#### AGEs and Melanoidins

**Figure 5:**
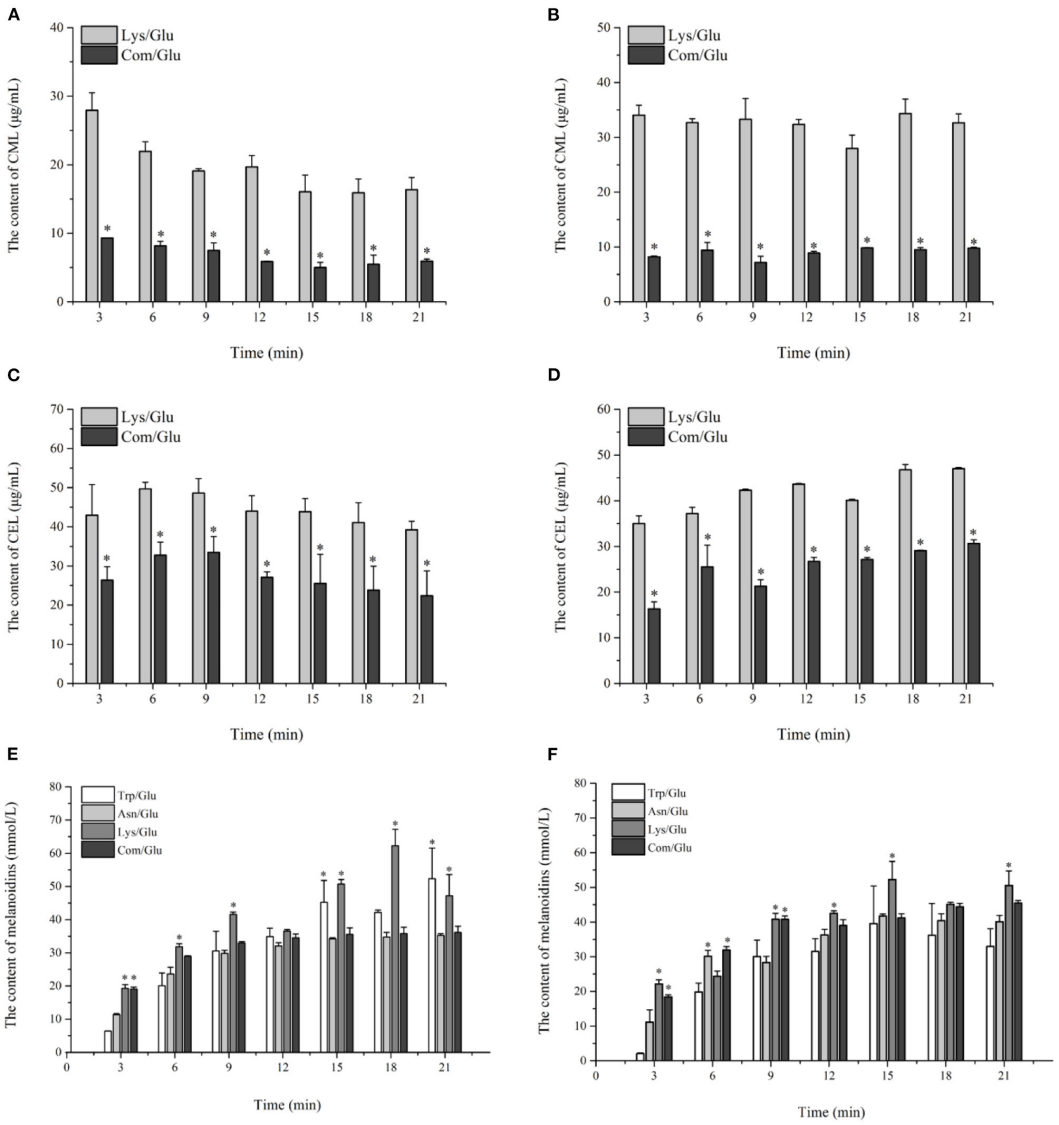
Kinetic profiles of Maillard reaction products includes CML **(A,B)**, CEL **(C,D)**, and melanoidins **(E,F)** in the different amino acid/glucose model system heated at 170°C **(A,C,E)** and 200°C **(B,D,F)**. Data were expressed as mean ± SD in triplicates (*n* = 3), *, (*P* < 0.05).

The trends for the two AGEs, CML and CEL, are shown in Figures 5A–D. Contrary to the results already presented for acrylamide and HAs, the amount of CML and CEL formed at the two reaction temperatures reached a maximum level in the initial reaction stage, and remained stable or decreased during the remainder of the reaction. The content range of CML produced in the Com/Glu and Lys/Glu models when heated to 170°C was from 5.01 ± 0.75 to 9.31 ± 0.02 μg/ml and from 16.35 ± 1.79 to 27.95 ± 2.54 μg/ml, respectively. When the heating temperature was 200°C, the content range of CML generated in the Com/Glu and Lys/Glu models was from 7.15 ± 0.14 to 9.80 ± 0.07 μg/ml and from 27.96 ± 2.42 to 34.34 ± 2.61 μg/ml, respectively. The content of CML formed in the Com/Glu model was significantly lower (*p* < 0.05) than that in the Lys/Glu model. In addition, the content of CEL produced in the Com/Glu model was also significantly lower than the content of CEL in the Lys/Glu model. The content range of CEL was from 22.40 ± 6.36 to 33.48 ± 4.02 μg/ml (for the Com/Glu model) and from 39.25 ± 2.17 to 49.67 ± 1.70 μg/ml (for Lys/Glu) at 170°C, while the ranges were from 16.31 ± 1.54 to 30.66 ± 0.79 μg/ml (for the Com/Glu model) and from 35.01 ± 1.66 to 46.99 ± 0.30 μg/ml (for the Lys/Glu model) at 200°C.

In addition to the already discussed MRHPs, melanoidins are generated via aldol condensations and the polymerization of carbonyl compounds and are considered as the main indicator of the browning state (35). The kinetic changes of melanoidins were also investigated in this study (Figures 5E,F). In general, the generation of melanoidins in all model systems exhibited a steep growth rate during the initial 9 min of heating, followed by a gentle increase during the remaining heating time. Specifically, the concentration of melanoids in the Lys/Glu and Trp/Glu models at 170°C (Figure 5E) were 47.20 ± 6.36 and 52.34 ± 9.23 mmol/L, respectively, which were significantly higher than the concentration of melanoids in the Asn/Glu and Com/Glu models. However, results differed when heating to 200°C (Figure 5F), as the concentration of melanoidins in the Trp/Glu model was significantly lower than that in the other model systems.

### Reaction Kinetic Modeling and Kinetic Parameters

According to available experimental data, and the reaction scheme proposed in Figure 1A. The kinetic data were fitted, and related kinetic parameters (k_1_-k_18_) were calculated by the Marquardt non-linear least squares regression.

A comparison of the kinetic parameter estimation among the four model systems is shown in Table 1. In general, the reaction rate of each time point increased with an increase in the reaction temperature, which is consistent with the general rule of kinetics (19). The formation of melanoids in all the model systems was the fastest. The formation of CML, CEL, and acrylamide (k_3_, k_4_, and k_5_) in the Com/Glu model system was significantly reduced when compared with the kinetics in the Lys/Glu and Asn/Glu model systems (*p* < 0.05). Previous studies also found that the addition of Lys to the Asn/Glu model can significantly reduce the production of acrylamide, but those studies did not consider the formation of CML and CEL (22, 36). These studies stated that the high activity of Lys in the MR was responsible for Asn's competition for the reducing sugar (22, 36). From Table 1, it can be seen that k_5_ was significantly higher than k_3_ and k_4_, thus indicating that acrylamide was formed more rapidly than CML and CEL in the simulation systems. According to previous research (18, 37), after the rapid formation of acrylamide, the presence of Lys could lower the acrylamide yield via adduct formation; this is because the electrophilic double bond of acrylamide can participate in nucleophilic reactions with hydrogen-bearing groups, such as the -NH_2_ group of Lys. This addition reaction not only causes a decrease in acrylamide, but may also reduce the generation of CML and CEL by consuming Lys. When Asn and Lys participate in the MR together, the reason for the significant decrease in the formation of acrylamide, CML, and CEL may be the competition for α-dicarbonyl compounds (especially GO), as Amrein et al. (18) reported that α-dicarbonyl compounds play a key role in the formation of acrylamide.

**Table 1:** Estimation of kinetic parameters among different amino acid model systems.

**Group/T (**°**C)**	**Kinetics parameters^a^**										
	${\text{k}}_{1}^{*}\;$	${\text{k}}_{2}^{*}\;$	${\text{k}}_{3}^{**}\;$	${\text{k}}_{4}^{**}\;$	${\text{k}}_{5}^{*}\;$	${\text{k}}_{6}^{*}\;$	${\text{k}}_{7}^{*}\;$	${\text{k}}_{8}^{*}\;$	${\text{k}}_{9}^{***}\;$	${\text{k}}_{10}^{***}\;$	${\text{k}}_{11}^{*}\;$
Lys-Glu/170°C	0.135	0.030	0.042	0.721	–	0.041	0.012	–	–	–	2.198
Trp-Glu/170°C	0.172	0.029	–	–	–	0.049	0.006	–	0.006	0.019	1.833
Asn-Glu/170°C	0.257	0.015	–	–	0.062	0.197	0.105	0.103	–	–	1.579
Com-Glu/170°C	0.199	0.023	0.015	0.523	0.050	0.114	0.072	0.071	0.012	0.015	1.420
Lys-Glu/200°C	0.178	0.029	0.072	0.441	–	0.027	0.014	–	–	–	2.369
Trp-Glu/200°C	0.199	0.029	–	–	–	0.045	0.005	–	0.007	0.063	2.480
Asn-Glu/200°C	0.143	0.037	–	–	0.095	0.237	0.114	0.112	–	–	1.864
Com-Glu/200°C	0.160	0.026	0.022	0.211	0.089	0.155	0.101	0.099	0.028	0.052	1.882

a Data were expressed as mean ± SD in triplicates (n = 3) and fit by the Marquardt non-linear least squares regression method. *, min^−1^; **, 10^−2^ min^−1^; ***, 10^−3^ min^−1^.

In addition, previous studies have reported that the addition of Trp to the Asn/Glu model can significantly reduce the production of acrylamide (22), but research in this area indicates that the main cause of acrylamide's significant decrease was due to Trp competing with Asn for reducing sugars, and a non-covalent interaction occurred between acrylamide and Trp (22).

According to the results of this study, the formation of acetaldehyde and acrolein (k_6_ and k_7_) in the Com/Glu model was significantly reduced compared with their formation in the Asn/Glu model. As a result, acrylamide (k_8_) produced by the acrolein oxidation pathway was also significantly reduced. In contrast, a significant increase in the content of harmane generated in the Com/Glu model was observed. As shown in Table 1, the formation of acetaldehyde and acrolein (k_6_ and k_7_) in the Com/Glu model increased significantly compared with its formation in the Trp/Glu model. According to Herraiz et al. (17), acetaldehyde is an important intermediate for the formation of β-carboline HAs. In the presence of Trp, acetaldehyde can be used to form THβC by Pictet–Spengler condensation, and then this will further generate harmane by the oxidation of THβC (Figures 1B,C). Therefore, Trp's and Asn's competition for acetaldehyde and thus the decrease in the production of acrolein may be an important reason for the higher content of harmane and lower content of acrylamide observed in the Com/Glu model compared with the Trp/Glu model.

## Conclusion

In order to illustrate the effect of multiple precursor amino acids on the simultaneous formation of multiple MRHPs in real heat-processed food systems, aqueous MR chemical model systems were established according to the concentrations and proportions of Lys, Asn, Trp present in real cereal and potato-based food. Changes in the content of precursors, MRHPs, key intermediates, and kinetic parameters in the MR model systems were determined. Our findings indicated significant interaction effects of Lys, Trp, and Asn in the MR model system. The competitive effect between Lys and Asn led to a significant reduction in CML, CEL, and acrylamide. Additionally, it could be explained that the formation of harmane is carried out through Pictet–Spengler condensation and the oxidation of THβC, which significantly increased the content of harmane, although the formation of acrylamide from the acrolein oxidation pathway decreased. Our results revealed the influence of multiple precursor amino acids on the formation of their MRHPs. These findings will be invaluable for understanding the simultaneous formation and interaction of multiple MRHPs in heat-processed food, will aid in the development of new processes for simultaneous control pathways, and help to improve the safety and quality of heat-processed food.

## Data Availability Statement

The original contributions presented in the study are included in the article/Supplementary Material, further inquiries can be directed to the corresponding authors.

## Author Contributions

YJ: formal analysis. CC and WQ: investigation. MZ: methodology. JC: project administration. ZH and QS: resources. WQ: writing—original draft. All authors contributed to the article and approved the submitted version.

## Funding

This work has been supported by the National Natural Science Foundation of China (Grant No. 3217160166), the Key Research and Development Plan of Guangdong Province, China (Grant No. 2019B020212011), and the National First-class Discipline Program of Food Science and Technology (No. JUFSTR20180201).

## Conflict of Interest

The authors declare that the research was conducted in the absence of any commercial or financial relationships that could be construed as a potential conflict of interest.

## Publisher's Note

All claims expressed in this article are solely those of the authors and do not necessarily represent those of their affiliated organizations, or those of the publisher, the editors and the reviewers. Any product that may be evaluated in this article, or claim that may be made by its manufacturer, is not guaranteed or endorsed by the publisher.
