# Supplementary material for: The Simultaneous Formation of Acrylamide, β-carbolines, and Advanced Glycation End Products in a Chemical Model System: Effect of Multiple Precursor Amino Acids
Source: Front Nutr. 2022 Mar 9;9:852717. doi: 10.3389/fnut.2022.852717 (PMC8959668; doi:10.3389/fnut.2022.852717)
Supplement: Supplementary file 1 [file Data_Sheet_1.docx]

**Supplement Table 1 Validation Parameters of the Proposed Method (Glucose and amino acids)**

| Compound | Linear range  (μg L^-1^) | Coefficients (r^2^) | LODs  (μg g^-1^) | LOQs  (μg g^-1^) | Spiked level  (μg g^-1^) | Mean recovery%^a^ (RSD%) |
| --- | --- | --- | --- | --- | --- | --- |
| Glucose | 4.45-1140 | 0.9692 | 0.17 | 0.55 | 45.6 | 87.1 (3.4) |
|  |  |  |  |  | 1140 | 83.1 (5.7) |
| Asn | 1.97-1008 | 0.9972 | 0.24 | 0.32 | 14.0 | 110 (5.9) |
|  |  |  |  |  | 504 | 110 (8.5) |
| Trp | 0.41-208 | 0.9989 | 0.29 | 0.38 | 2.9 | 118 (7.1) |
|  |  |  |  |  | 104 | 89.9 (13.4) |
| Lys | 1.98-1012 | 0.9981 | 0.26 | 0.34 | 2.9 | 116 (6.2) |
|  |  |  |  |  | 104 | 96.6 (13.6) |

^a^ Intraday and interday precisions are given in parentheses (n = 3).

**Supplement Table 2 Validation Parameters of the Proposed Method (MRPs and intermediates compounds)**

| Compound | Linear range  (μg L^-1^) | Coefficients (r^2^) | LODs  (ng g^-1^) | LOQs  (ng g^-1^) | Spiked level  (μg g^-1^) | Mean recovery%^a^ (RSD%) |
| --- | --- | --- | --- | --- | --- | --- |
| Acrylamide | 0.26-21.0 | 0.9998 | 72.1 | 241 | 0.42 | 88.2 (10.7) |
|  |  |  |  |  | 2.10 | 89.9 (14.6) |
| Harman | 0.007-1.01 | 0.9969 | 1.49 | 4.97 | 20.1 | 98.7 (15.3) |
|  |  |  |  |  | 100 | 109 (9.9) |
| Norharman | 0.008-1.07 | 0.9905 | 1.08 | 2.93 | 21.4 | 97.8 (8.3) |
|  |  |  |  |  | 106 | 105 (9.2) |
| CML | 0.04-2.49 | 0.9994 | 9.35 | 6.23 | 1.24 | 90.3 (8.2) |
|  |  |  |  |  | 7.48 | 120 (3.6) |
| CEL | 0.07-3.63 | 0.9993 | 8.65 | 5.77 | 1.15 | 109 (14.6) |
|  |  |  |  |  | 6.92 | 123 (5.8) |
| Ethanal | 0.19-50.0 | 0.9985 | 13.9 | 46.5 | 0.78 | 100 (1.7) |
|  |  |  |  |  | 12.5 | 90.1 (4.6) |
| Acrolein | 0.31-10.0 | 0.9991 | 83.2 | 277 | 0.20 | 114 (10.2) |
|  |  |  |  |  | 5.00 | 99.4 (3.6) |
| MGO | 0.37-23.4 | 0.9998 | 60.5 | 201 | 0.29 | 104(3.9) |
|  |  |  |  |  | 6.92 | 93.0 (5.0) |
| GO | 0.39-24.9 | 0.9991 | 117 | 373 | 0.40 | 108 (9.4) |
|  |  |  |  |  | 6.30 | 108 (2.9) |

^a^ Intraday and interday precisions are given in parentheses (n = 3).

**
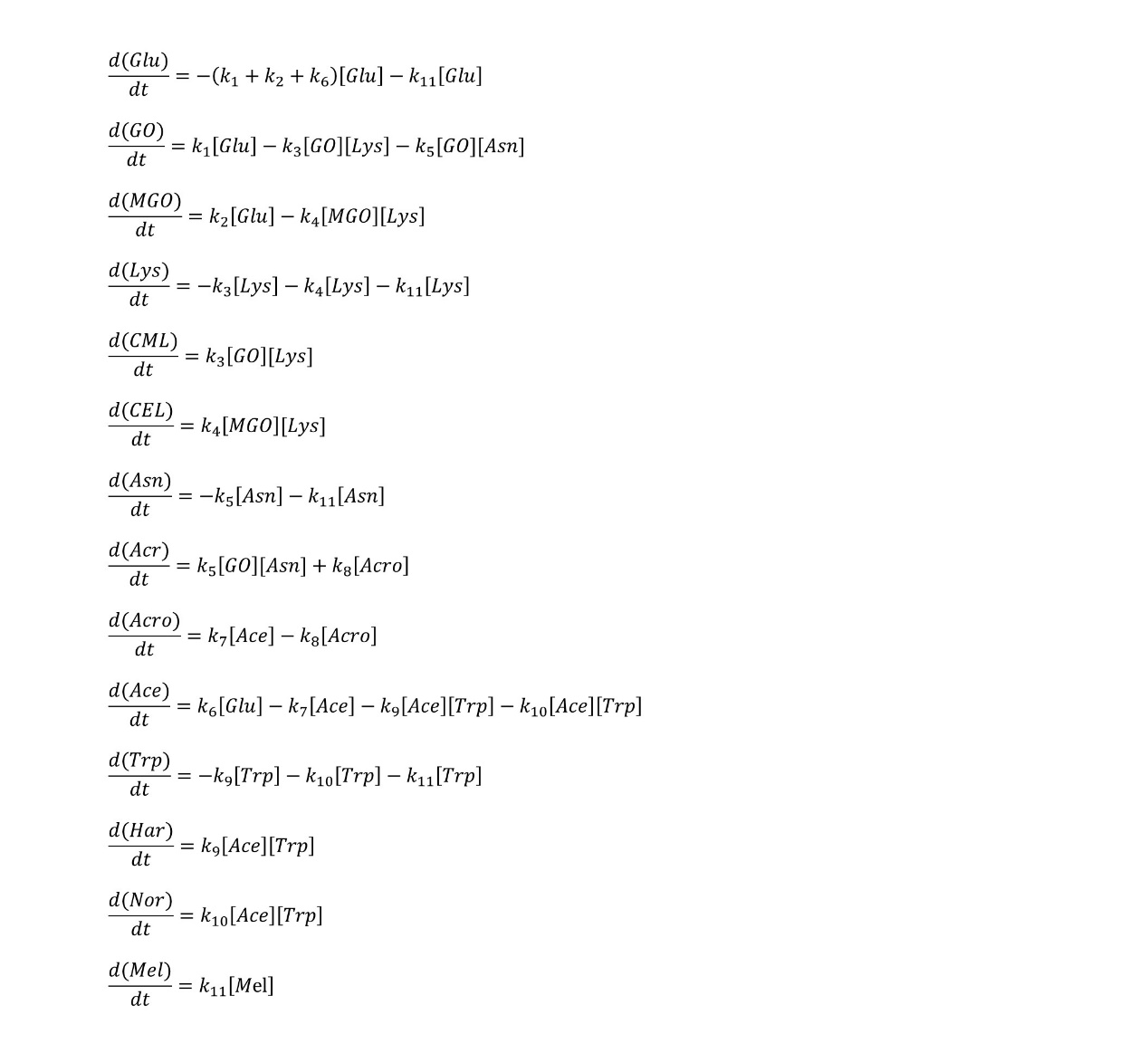
**

**Supplementary Figure 1** Rate equations derived from the kinetic mechanism. [Glu] = glucose, [Lys] = lysnine, [AAs] = amino acids, [Acr] = acrylamide, [Go] = glyoxal, [MGO] = methylglyoxal, [Trp] = tryptophan, [AP] = amadori product, [Acro] = acrolein, [Ace] = acetaldehyde, [Asn] = asparagine, [CML] = NƐ-(Carboxymethyl)lysine, [CEL] = NƐ-(Carboxyethyl)lysine, [Har] = harman, [Nor] = norharman, [Mel] = melanoidins. *k*_1_- *k*_11_ = rate constants.
